# Methods of green synthesis of Au NCs with emphasis on their morphology: A mini-review

**DOI:** 10.1016/j.heliyon.2021.e07250

**Published:** 2021-06-09

**Authors:** Khali Sayadi, Fatemeh Akbarzadeh, Vahid Pourmardan, Mehdi Saravani-Aval, Jalis Sayadi, Narendra Pal Singh Chauhan, Ghasem Sargazi

**Affiliations:** a Young Researchers Society, Shahid Bahonar University of Kerman, Department of Chemistry, Kerman, Iran; b Department of Microbiology, Islamic Azad University Kerman, Kerman, Iran; c Department of Environmental Engineering, University of Zabol, Zabol, 98613-35856, Iran; d Young Researcher, Department Environmental Engineering, University of Zabol, Zabol, 98613-35856, Iran; e Young Researchers Society, Zabol University of Medical Sciences, Zabol, Iran; f Department of Chemistry, Faculty of Science, Bhupal Nobles' University, Udaipur, 313002, Rajasthan, India; g Noncommunicable Diseases Research Center, Bam University of Medical Sciences, Bam, Iran

**Keywords:** Au nanostructures, Green synthesis, Intracellular, Extracellular

## Abstract

Greener synthetic methods are becoming more popular as a means of reducing environmental pollution caused by reaction byproducts. Another important advantage of green methods is their low cost and the abundance of raw materials. Herein, we investigate the green Au nanoclusters (NCs) using microorganisms (bacteria and fungi) and plant extraction with various shapes and development routes. Natural products derived from plants, tea, coffee, banana, simple amino acids, enzyme, sugar, and glucose have been used as reductants and as capping agents during synthesis in literature. The synthesis techniques are generally chemical, physical and green methods. Green synthesis of Au NCs using bacteria and fungi can be divided into intracellular and extracellular. In an intracellular manner, bacterial cells are implanted in a culture medium containing salt and heated under suitable growth conditions. However, in an extracellular manner, the Au ions are directed from the outside into the cell. Thus, these methods are considered as a better alternative to chemical and physical synthesis. The research on green synthesis of Au nanoparticles (NPs) and its influence on their size and morphology are summarized in this review.

## 1 Introduction

Several engineered Au nanostructured materials have been widely explored in biomedical fields and catalytic activities [1]. For centuries, gold has been recognized as a nontoxic, safe inorganic agent. Au has a lot of potential in biological and catalytic applications, especially when it comes in the form of nanoparticles [2]. Many efforts have been made to develop new, more environmentally sustainable methods of producing nanoparticles [3]. In comparison to other synthesis techniques that produce hazardous by-products from harmful reductive organic species, the motivation has been to use less harmful synthesis methods [4, 5].

Because of their biocidal properties and wide range of applications, Au-based compounds have been used as nontoxic inorganic agents in many countries. Au and its related compounds are non-toxic to animal cells but toxic to microorganisms such as bacteria and fungi. The recent advances in the synthesis of Au-NPs have a significant impact on a wide range of scientific fields. The unique properties of these nanoparticles have prompted numerous studies and applications in electronics, nanomedicine, biomaterials, energy, and food. Au-based compounds are now recognized as an important class of nanomaterial, primarily used as catalysts or antibacterial/antifungal agents.

Nowadays, the application of nanotechnology has played a key role in the development of science. Nano-science has shown that if the material size is varied to be nanometer [6], unique characteristics such as localized surface plasmon resonance (LSPR) [7], electrical conductivity [8], hardness [9] and chemical reaction [10] can be achieved very differently. Also, With the spread of Corona virus [11] and other dangerous sickness [12, 13], it is becoming increasingly important to application of nanotechnology [14, 15, 16].

Metallic nanoparticles (MNPs) are widely applied because of their small size [17], the proliferation atoms on the surface (high surface area to volume ratio) [18] and excellent reactivity [10]. Also, the effects of the MNPs should be predictable, controllable, and obtain optimal results with minimal toxicity while being biocompatible [19, 20].

There are three main methods for preparing MNPs: chemical, physical and green chemistry approaches. According to the chemical process, metal ions are converted to metal using reducing and stabilizing agents such as sodium borohydride, sodium citrate, polyols, alcohols, sodium dodecyl sulfate etc [17]. With the progress of different physical and chemical methods for the preparation of MNPs, toxic environmental pollution problems have been enhanced [18, 19, 20, 21]. These procedures appear to generate dangerous products that can directly harm the environment. For this reason, the synthetic approach of environment-friendly green chemistry has been proposed as a good alternative [22].

The green synthesis has no high pressure, energy, toxic materials, temperature and eco-friendly [23, 24]. As a result, many researchers are attempting to synthesize MNPs using biological systems. Microorganisms and plant extraction are used in the fabrication of MNPs with green chemistry because they are generally inexpensive, readily available, and nontoxic [25, 26]. Fatty acids, aromatic compounds, protein, polysaccharides, and other active biomolecules found in plants and microorganisms make it easier to reduce metal ions [27]. Among MNPs, Au NCs have a high potential for use in biology and medicine due to control of surface chemistry with different functional groups, adjustment of size, geometric shape, unique optical performance, easy green synthesis, a high surface area, and high surface energy, to provide stability and immobility of biological molecules for their biological activity [28, 29, 30, 31, 32, 33, 34]. Bio-synthesis of Au NCs can be used in various applications such as antimicrobial activities, nano-bio sensors for diagnosis and therapy, drug delivery and adsorption [35]. Microorganisms such as bacteria, yeasts, fungi, and algae were used in the biosynthesis of metal nanoparticles in a variety of ways. The use of plants to make metal nanoparticles has recently sparked a flurry of green synthesis research. This review will provide a quick overview of current research on green Au nanoparticle synthesis and explain how different synthesis methods affect the size and morphologies of Au NCs.

## 2 Au NCs synthesis using microorganisms

The green synthesis methodology of Au NCs through microorganisms includes a wide range of bacteria and fungi [35, 36]. The important advantages of bio-synthesis methods of Au NCs compared to chemical approaches is the lack of pollution, facile synthesis of single-stage and biocompatibility. Besides, no external stabilizing and reducing agents are needed in this type of synthesis because the bioactive compounds in organisms perform these roles well. These agents convert the Au^3+^ into Au^0^ [37, 38, 39, 40]. Accordingly, these strategies allow to prevent the aggregation and agglomeration of Au NCs as well as other NCs and provide a suitable capping for the production process and their stability [39, 40, 41].

### 2.1 Bacteria

In the production of Au NCs using microorganisms, bacteria compared to other microorganisms are highly regarded. One reason for preferring bacteria to synthesize Au NCs is their ease of access and the possibility of increasing genetic changes [42, 43]. Bacteria resist against heavy metals carried out with diffusion phenomena from inside to outside the cell. The roles of diffusion and detoxification of metals are the responsibility of membrane proteins, which act as reverse transporters of cations and protons or ATPase. Thus, microbial systems can convert toxic inorganic ions into nontoxic and soluble ions by regeneration and precipitation [44]. Bacteria can target ion from the reaction solution and then regenerate it. Depending on the reduced metal ion form's accumulation location, nanoparticle synthesis will be of two types: intracellular or extracellular [44].

#### 2.1.1 Intracellular

**Figure 1:**
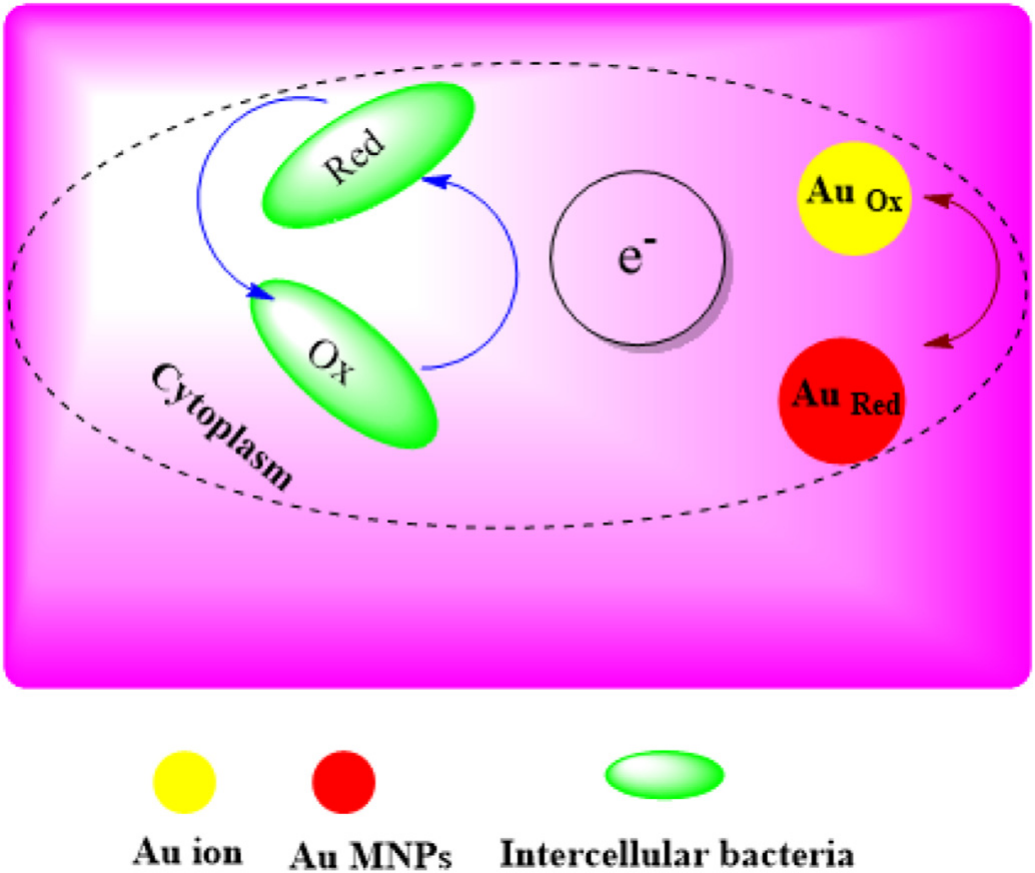
Schematic mechanism of intracellular bacterial synthesis of Au MNPs [37].

Bacteria that have an Au-resistant system can biosynthesize Au NCs intracellular, provided that the concentration of Au ions is not higher than their tolerance range. Also, bacteria that create Au NCs in this way are sensitive to high concentrations of Au ions. In other words, Au has a dual performance, in which it stimulates bacteria to manufacture nanoparticles at low concentrations and induces cell death at higher concentrations [45]. In intracellular construction, bacterial cells are implanted in a culture medium containing salt and heated under suitable growth conditions. Subsequently, ions are transported in and altered Au NCs using the intracellular enzymes and other factors [46, 47] (Figure 1).

For the intracellular synthesis of Au NCs, the use of ultrasound or other required reactions compatible with appropriate detergents is helpful. For this purpose, recovering precious metals in mineral wastewater and biosynthesis of Au NCs is helpful. Mann has reported Intracellular synthesis of octahedral Au NCs with a size range of 5–25 nm using *Pedomicrobium* bacteria [48]. Some bacteria tend to form membrane vesicles to protect themselves against toxic chemical structures. A sample of these bacteria, is *P. boryanum UTEX485* cyanobacteria, was reported by Lengke *et al.* They examined the preparation of Au NCs in the presence of two solutions, Au(S_2_O_3_)_2_^−3^ and AuCl_4_^−^. The results showed that in the presence of cyanobacteria and Au(S_2_O_3_)_2_^−3^ solution, the morphology Au NCs is cubic with a size of 10–25 nm, in contrast to the presence of AuCl_4_^−^ solution, it is octahedral and <10 nm [49, 50]. Recently, Du *et al.* reported the synthesis of spherical, hexagonal, and trigonal Au NCs utilizing *Escherichia coli* for electrochemical applications [51]. Table 1 summarizes other types of intracellular synthesized NCs morphologies. Research has been conducted on the role of environmental conditions such as pH and temperature, the intracellular synthesis of Au NCs and, their impact on morphology.

**Table 1:** List of bacteria that intercellular synthesize Au NCs.

Bacteria	Size	Morphology	References
*Bacillus subtilis 168*	5–25 nm	Octahedral	[52]
*Shewanella algae*	10–20 nm	Periplasmic	[53]
*Plectonema*	10 nm	Cubic	[49, 50]
*Rhodobacter*	Not determine	Spherical	[54]
*Delftia sp*	11.3 nm	Spherical	[55]
*Bacillus megaterium MSBN04*	Not determine	Not determine	[56]
*Halomonas salina*	30–100 nm	Spherical	[57]
*Deinococcus. R*	43.75 nm	Spherical, triangular, Irregular	[58]
*Marinobacter algicola*	74 nm	spherical, cubical, triangular, Penta-hexagonal	[59]
*Enterococcus sp.*	Not determine	Spherical	[60]
*Geobacillus sp. strain ID17*	5–50 nm	Quasi-hexagonal	[61]

It was then suggested that in the different results from the published works in the intracellular, particle morphologies synthesized using bacteria ranged from cubic, periplasmic, irregular, triangular, penta, spherical, triangular, octahedral, and quasi-hexagonal. The first synthesis involving bacteria-mediated approaches for metal nanoparticle synthesis was conducted at the turn of the twentieth century. Au NPs with a 15 ± 10 nm diameter in octahedral shape were synthesized using *Bacillus subtilis 168* [52]. In previous studies involving algae, *Shewanella algae* isolated were able to produce Au NPs within the periplasmic space with a diameter of 15 ± 5 nm [53]. The mechanism of nanoparticle formation in the case of bacteria-based synthesis was that in the first step, Au ions could adsorbed on the surface of the mycelia and not in the solution due to the electrostatic interactions between negatively charged cell wall of mycelia and positively charged Au ions. Finally, the Au ions are reduced by enzymes found in cell walls, resulting in the formation of Au nuclei. A balanced distribution of size and lack of nanoparticle accumulation is one of the essential characteristics of nanoparticles in different ambient conditions such as microorganisms, bacteria culture conditions, pH, and temperature effects. Kumar *et al.* evaluated *Delftia sp. strain KCM-006* bacteria for the synthesis of Au NCs. In this study, based on two factors, pH and temperature, synthesized spherical Au NCs with a size of 11.3 nm were obtained. In fact, during the reaction, the pH and temperature changed, and by optimizing the pH = 8 and the 450 °C, these NCs were obtained [55]. Synthesis using *Rhodobacter*, *Rhodobacter*, and *Enterococcus sp.* take the green approach ever further, which led to spherical shape [54, 56, 60]. In another interesting study, Shah *et al.* reported anisotropic and non-anisotropic AuNCs in pH = 4 and pH = 9, respectively, using *Halomonas salina*. This reaction's proposed mechanism is that probably NADH (Nicotinamide adenine dinucleotide) enzyme acts as a reducing agent. In acidic pH, proton ions' concentration increases, and functional groups in the NADPH (Nicotinamide adenine dinucleotide phosphate) have a positive charge. As a result, an unstable Au NCs are created. Conversely, in alkaline pH, the reducing agent potential of NDPH increases, and thermodynamically, stable nanostructures are formed [57].

#### 2.1.2 Extracellular

**Figure 2:**
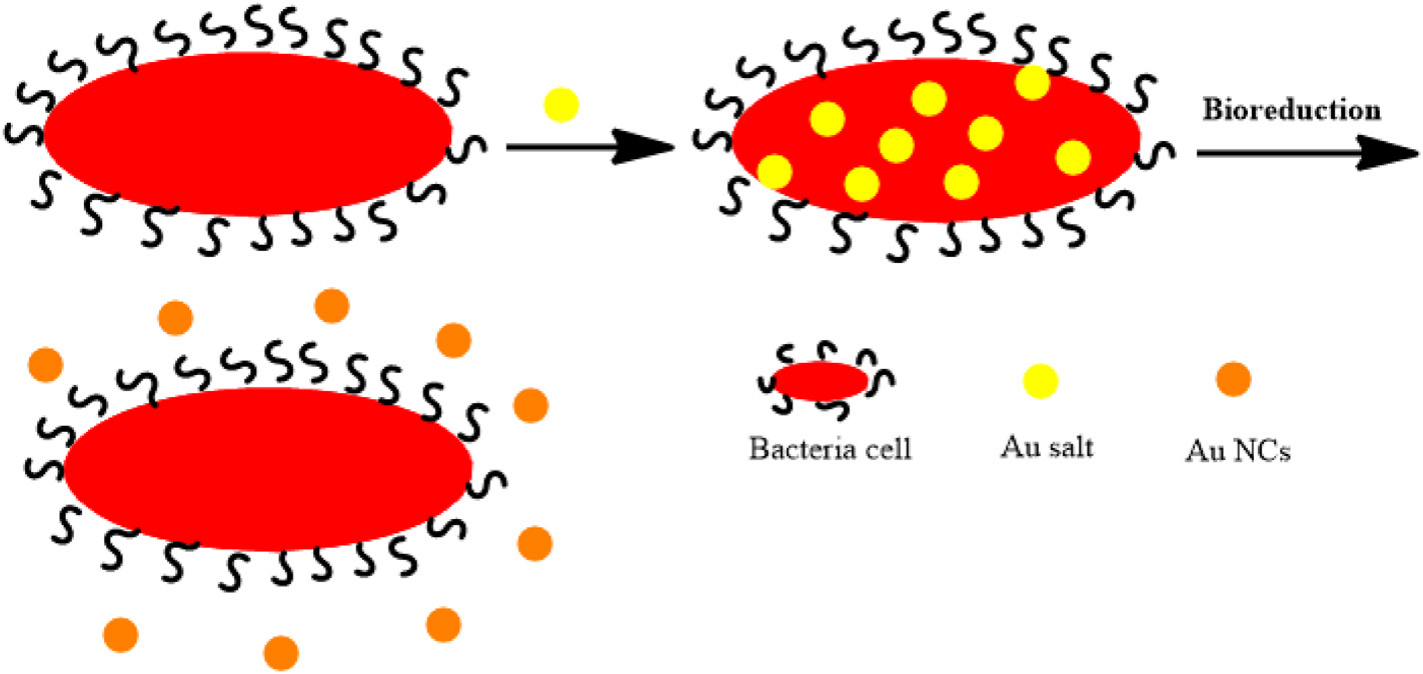
Extracellular synthesis of Au NCs [53].

The extracellular synthesis strategy (Figure 2) of Au NCs is performed in a variety of forms, such as spherical, disc-like, cubic, hexagonal, and triangular (Table 2), with synthetic bacterial methods such as biomass, bacterial culture supernatant (BCS) and cell-free extract (CFE) [34, 62].

**Table 2:** List of bacteria that extracellular synthesize Au NCs.

Bacteria	Size	Morphology	References
*B. niabensis 45*	10–20 nm	Spherical	[67]
*Bacillus licheniformis*	38 nm	Spherical	[68]
*Marinobacter Pelagius*	10–20 nm	Spherical, Triangular	[69]
*Stenotrophomonas malophilia*	40 nm	Oval	[38]
*Cupriavidus metallidurans* CH34	15.5 nm	Triangular, Decahedral	[70]
*Nocardiopsis sp.*	11.57 ± 1.24 nm	Spherical	[71]
*R. sphaeroides*	10 ± 3 nm	Spherical	[72]
*L. casei*	26.9 nm	Not determine	[73]

#### 2.1.3 Biomass

When a type of bacterial cell biomass is exposed to Au salt, it can generate Au NCs. The bacterial cell is capable of manufacturing Au NCs that, in some cases, can be secreted outside the cells. Besides it, if the NCs resulted are located outside the bacterial cell, the NCs are synthesized extracellularly. In the first case, the bacterium secretes the Au NCs produced through the intracellular to the outside of the cell. Some of these NCs may be attached to the cell wall, which can be separated by ultrasonic waves. In contrast, in the second case, biological molecules secreted outside the cell regenerate Au ions to establish Au NCs [62].

#### 2.1.4 BCS

The microbial culture medium is regenerative due to the presence of compounds such as tryptone and yeast extract, and it is useful alone for the biosynthesis of NCs [63]. In the BCS route, chemicals produced by bacteria secreted outside the cell in the culture medium are considered effective levers, and no bacterial cells are needed. Regarding this method, after culturing the bacteria for 24–48 h in the liquid culture medium, the composition containing culture medium and the bacterium is centrifuged, and the supernatant liquid is collected. In ideal conditions, the supernatant liquid is exposed to Au salt to synthesize Au NCs. The role of organic matter and cell culture compounds is to regenerate Au ions to Au NCs. The most important problem with this method is the synthesis of Au NCs surrounded via organic compounds in the culture medium. Therefore, it disrupts the dispersion, recovery, and properties of Au NCs, and is less commonly used in conventional applications [64].

#### 2.1.5 CFE

CFE synthetic mechanism is suggested as a good alternative to biomass and BCS methods to overcome the latter ones' problems. Based on this technique, the bacterial biomass is incorporated with water and placed in favorable conditions for 24–72 h. The biomass and compounds of the culture medium are then washed and removed. In this way, the biological molecules produced by the bacteria, which are released due to cell lysis or enduring undesirable conditions, can synthesize Au NCs [65].

## 3 Fungi

**Figure 3:**
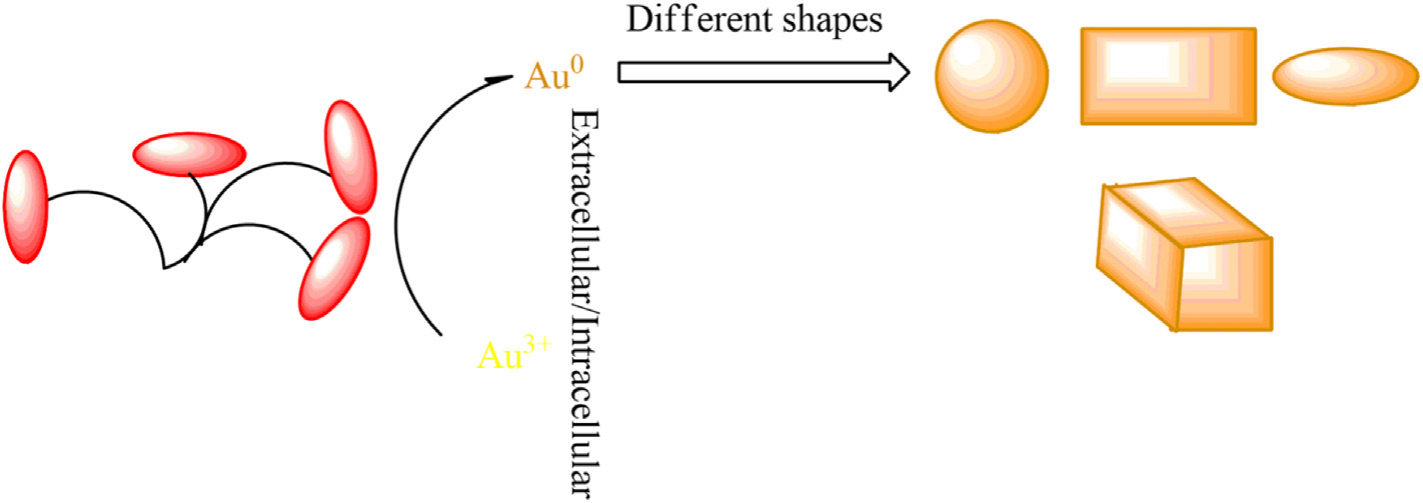
Schematic illustration of the extracellular/intracellular biosynthesis of Au NCs with [66].

Fungi are less noticeable than bacteria and plant extracts because their microscopic and mechanical properties make it difficult to characterize metal NCs [66]. These microorganisms have several advantages over the biosynthesis of Au NCs, including large amounts of extracellular enzymes, larger size distribution, and protein secretion into the extracellular space compared to other microorganisms [66]. Having a secretome factor plays a special role in reducing and capping the bio-fabrication of Au NCs. In extracellular synthesis, Au^3+^ ions are trapped in the fungus' cell wall and are converted Au NCs through bio-structures such as enzymes and proteins. In intracellular strategy, fungi also penetrate Au^3+^ ions with cell membranes' help and then convert their redox intermediates into Au NCs [66]. Figure 3 shows the intracellular/extracellular synthesis of NCs. The extracellular approach is involving *B. niabensis 45* bacteria for the metal nanoparticle synthesis was performed with a diameter of 15 ± 5 nm in spherical shape [67]. Synthesis using *Bacillus licheniformis* takes the green approach. During exposure of the bacteria to the solution, the reduction of ions and the formation of Au NPs occurred with a diameter of 38 nm and spherical shape [68]. It was then suggested that the different results of the extracellular approach using bacteria ranged from spherical, triangular, decahedral, and oval shaped Au NPs from the published works. In the case of bacteria-based synthesis, the nanoparticle formation mechanism was that the secreted enzymes are responsible for the reduction process. The advantage of extracellular synthesis is that the nanoparticles produced will not bind to the biomass. As a result, this approach can be extended to the biosynthesis of nanomaterials with a variety of chemical compositions, such as oxides and nitrides.

In the generation of Au NCs by intracellular method, distribution and diffusion into the fungal cell membrane usually occur. Also, the smaller Au^3+^ ions than the Au ^+^ ions makes it easier to penetrate and diffuse into the cell [74]. Nevertheless, the two parameters of aggregation and absorption are considered two disturbing factors. If the release of Au ions into the cell is carried out with these two, it is not stable. There is a particular relationship between the growth and expression of proteins and Au NC's concentration in *Rhizopus oryzae*. As the concentration of these NCs increases, the ratio of Au^3+^/Au^+^ decreases, thereby increasing the growth and expression of proteins is exhibited [75]. Sheikhlou *et al.* used *Phoma macrostoma* to biosynthesis Au NCs. Mixing the fungus with an aqueous solution of Au ions results in a change from yellow to purple, indicating Au NCs. Synthesized NCs with 100–200 nm were obtained with spherical, triangular, and rod shapes [76]. The potential of Au synthesis NCs using fungi can be used against pathogens. Marwa Abdel-Kareem *et al.* evaluated the antibacterial activity of Au NCs, and this work used *Trichoderma hamatum* fungi to synthesize extracellular NCs. Bacteria were included *Bacillus subtilis ACCB 133*, *Staphylococcus aureus ACCB 136, Pseudomonas aeruginosa ACCB 156,* and *Serratia sp.* NCs synthesized under optimal conditions (pH = 7 and T = 38 °C), which their uniform size was reported to be 5–30 nm with spherical, hexagonal, and pentagonal morphologies [77]. *Zhu et al.* were confirmed to be efficient to intracellular produce Au NCs with various uncontrolled shapes and sizes utilizing *Pycnoporus sanguineus* and its protein extraction. To improve the quality of the Au NCs, the protein extraction of *Pycnoporus sanguineus* were graded into five fractions by ultrafiltration (UF). In protein extraction compared to other fractions, obtained Au NCs with a size <30 nm have efficiency above 90% and 87% via spherical morphologies and optimized conditions (pH = 4, 30 °C, and 0.5 mM AuCl_4_ －), Au NPs have a narrower distribution with the size of 6–25 nm. Accordingly, this mechanism can be explored in the absorption of various functional groups on a substrate such as amines and carboxylic, resulting in the formation of spherical Au NCs with small size [78]. Also fungi of *Aspergillus clavatus* (Extracellular, 24.4 ± 11 nm, triangular, spherical and hexagonal), *Neurospora crassa* (Intracellular, 32 nm, spherical), *Epicoccum nigrum* (Intracellular and extracellular, 5–50 nm, not determine), *Penicillium sp.* (Intracellular, 40–60 nm, spherical), *Marinobacter Pelagius* (Extracellular, 2–10 nm, spherical and triangular), Metal-tolerant fungal isolates (Intracellular, 9–18 nm, Spherical, trigonal, cubic, tetragonal and hexagonal), Rhodopseudomonas capsulate (Extracellular, 10–20 nm, spherical), *Yarrowia lipolytica* (Intracellular, not determine, related to Au^3+^ concentration), *Fusarium oxysporum* (Extracellular, 22–30 nm spherical and hexagonal), etc. are reported [79, 80, 81, 82, 83, 84, 85, 86, 87].

## 4 Plant extraction

**Figure 4:**
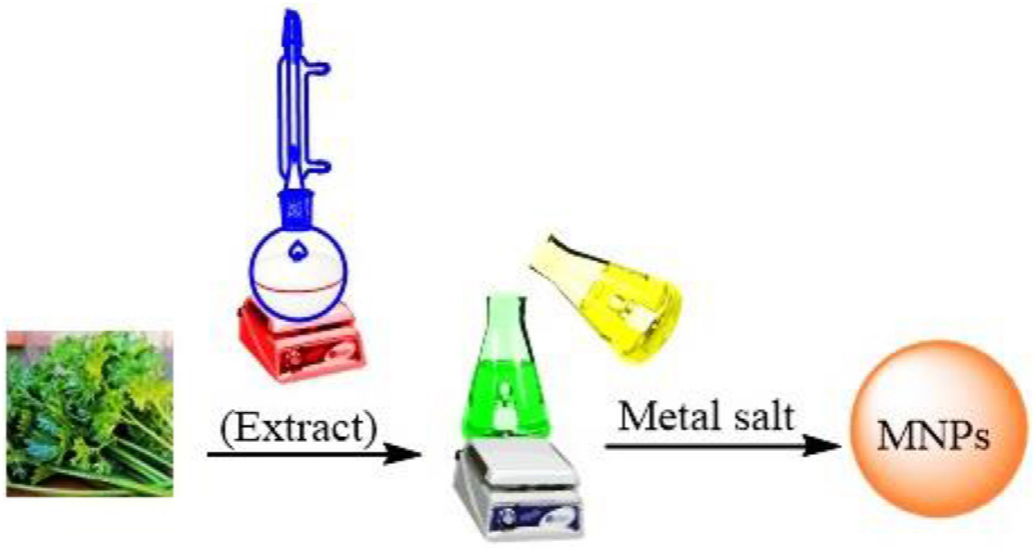
Preparation of MNPs by plant extraction method [81].

A general way of manufacturing MNPs, one of the plant parts such as seed, leaf, roots, stem, etc. is dried at room temperature and extracted. They are then mixed with a metal salt solution. Finally, during the reaction, the metal ion is converted to MNPs using reducing and stabilizing agents [88, 89] (Figure 4). MNPs of Au, Ag, Pd, Cu, ZnO, Pd, Fe, etc. have been synthesized by this technique [90].

The type and nature of the plant, temperature, pH, metal salt, and time, including factors that affect the generation of MNPs in this synthesis method [91].

**Figure 5:**
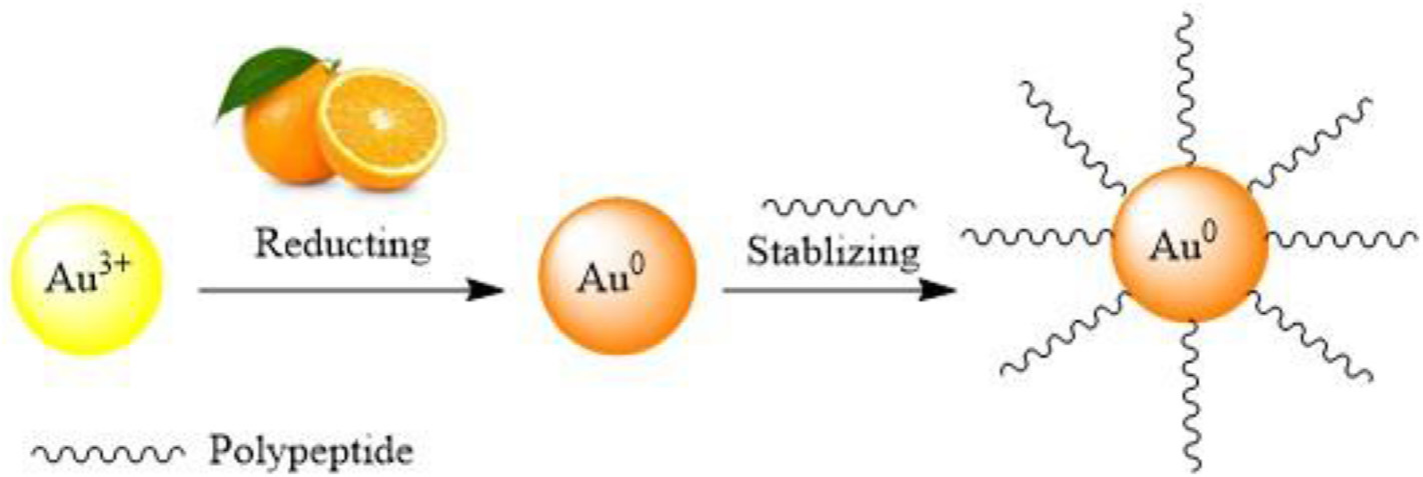
Synthesis of Au MNPs using *Citrus maxima* fruit extraction [93].

Jamrose et al. extract of three plants of *S. officinalis*, *M. officinalis,* and *M. piperita* were used to synthesize Au MNPs. The average size of spherical MNPs prepared for *S. officinalis* 15.1 ± 10.2 nm, *M. officinalis* 19.5 ± 24.3 nm, and *M. piperita* 55.1 ± 48.4 nm have been reported, respectively [92]. Yu *et al.* studied the synthesis of Au MNPs using aqueous extracts of *Citrus maxima* fruit with the size of the average particle of 25.7 ± 10 nm [93] (Figure 5).

Anuradha *et al.* evaluated different ratios of *Azadirachta indica* extract and [HAuCl_4_]^-^ to form Au MNPs. The results exhibited that their peak plasmon was stabilized at 556–558 nm. The reaction was complete in 24 h as soon as different ratios were added to each other, and the brick red Au MNPs were obtained [94]. Kashturi *et al.* reported the production of hexagonal and trigonal of Au MNPs with the enhanced concentration of [HAuCl_4_]^-^ and extraction of *Phyllanthus amarus* plant [90]. In other work, Liu *et al.* evaluated the antioxidant properties of Au MNPs, applying *Chrysanthemum* extract [95]. The apiin present in banana leaf extract demonstrated the potential of ions reduction to Au MNPs, a method reported by Kashturi *et al.* The reducer apiin produces with the generation of complexion through salt metal; as a result, Au MNPs was produced [96]. The green synthesis of Au MNPs using an extract of *Mentha piperita* showed considerable ability in antimicrobials, such as clinically isolated human pathogens carried out by Ali *et al.* [97]. The extract of *Ficus carica* was used to obtain Au MNPs. For the purpose, the studies conducted by specifications of synthesized materials were described [98]. Banoee *et al.* also reported the ethanol extract of *black tea* and *tannin* to synthesize Au MNPs. The role of ethanol during synthesis is reducing and stabilizing agent. The average size for ethanol extract of *black tea* and its ethanol-free *tannin* extract was 10 and 3 nm, respectively.

In contrast, no aggregation was observed for Au colloidal NPS gained by ethanol-free tannin at different time ranges [99]. Nagaraj et al. reported the synthesis of Au NPs using *Frangipani* flower. Two special advantages are included to encapsulate the Au MNPs. The other one is the reducing agent. During the fabrication of Au MNPs, due to their specific properties (SPR), their color varied from pale yellow to dark brown [100]. Dubey *et al.* synthesized Au MNPs using *Rosa rugosa*.

The functional carbonyl groups were importantly responsible for reducing metal ions to fabricate NPs, and FTIR spectroscopy confirmed this operation. Also, when the reaction's pH diminished, the size of the resulting NPs was 50–250 nm [101]. Another source to synthesize the Au MNPs is the extract of *Benincasa hispida* seed. During the reaction process, the carboxylic group (COOH) alters to carboxylate anion (COO^−^). The COO^−^ group could act as a surfactant to anchor on the surface of Au MNPs and stabilize through electrostatic interaction. The ambient conditions, such as temperature and pH, could be easily affected by particle size. So, Au NPs are stable in the range from 10 to 30 nm at pH = 6 [102]. Au MNPs were also produced utilizing *Justicia glauca* [103], *Alfalfa* [104], *Capsicum annuum L.* [105], *Commiphora wightii* [106], *Crinum macowanii* [107], *Hibiscus sabdariffa* [108], *Flammulina velutipes* [109], *Dracocephalum Kotschyi* [110], *Solidago Canadensis* [111], *Pseudomonas monteilii* [112], *Justicia adhatoda* [113] *Hamelia patens* [114], *Pongamia pinnata* [115], *Cucurbita pepo L* [116], *Aconitum violaceum* [117], *Ulva armoricana* [118], *Solanum lycopersicon* [119], *Annona squamosal* [120], *Sasa borealis* [121], *Prosopis farcta* [122] etc.

Because of the superior properties of nanocomposite materials over individual particles, research into the understanding and characterization of these materials has grown in importance [123]. After the reaction, to form a solvation sphere before they reach the frozen reactor walls. After the reaction, nanoparticles are warming at room temperature to form metal colloids. Depending on the metal concentration, metal type, organic solvents, and delay time to stabilize the colloidal nanoparticle, the nanoparticles' aggregation produce in different shapes, including spherical, clusters, and fractals. Undeniably, numerous selective homogeneous catalysts and medicinal applications from Au NCs have been reported. The only feature is the penetration of the reagents for the desired purposes [124, 125]. However, poor control on developing required and suitable size and morphology of Au nanoparticles in plants mediated synthesis for their potential applications has been a great contest that has attained the special focus of the research community to enhance the efficacy of biosynthesis protocol. The literature has revealed few research efforts for developing Au nanoparticles' different sizes and morphology with their further utilizations in catalytic applications by manipulating the reaction conditions [126]. Aromal and coworkers described the synthesis of smaller Au NCs, which acquired only 7 min to complete the photocatalytic reduction reaction [127].

On the contrary, larger size Au NCs took 712 min for completion of the photocatalytic reduction reaction. Moreover, Schimmel and coworkers reported the higher photocatalytic activity of smaller-sized Au nanoparticles that other nanoparticles contained a bigger size [128]. Rajan *et al.* stated spherical formation shaped Au NCs at elevated reaction temperature via higher volume, which exhibited better photocatalytic efficiency through the completion of photocatalytic reduction reaction in just 20 min. The anisotropic shape of Au NCs formed at low reaction temperature using the least volume, took 60 min to complete photocatalytic reduction reaction [126]. Therefore, it is highly stimulating to produce Au NCs with controllable size and shape by manipulating the reaction parameters to enhance the photocatalytic performance in various progressively growing industrial applications.

## 5 Conclusion

Many efforts have been made in recent decades to develop new green synthesis methods. Living organisms have enormous potential for producing nano-materials that can be used in a variety of fields, particularly biomedicine. Organisms extracted from plants ranging from simple bacteria to highly complex organisms can all be used to create nano-objects of the desired size and shape. Au NCs is now developed toward green synthesis to reduce toxic chemicals and it is compatible with the environmental protocols. The bioactive compounds from microorganisms such as bacteria, fungi and plants are proposed instead of toxic chemicals to produce safer and more biocompatible Au NCs for different applications. However, compared to others, the cultivation of the bacteria and large-scale production remains a challenge. As a first step, bacteria were investigated as the first nano factories capable of producing noble metal nanoparticles. However, due to the low synthesis rate and the limited number of size and shape distributions available, the research was limited to fungi and algae.

Fungi are an excellent choice for large-scale green nano production. They are simple to handle during downstream processing and secrete a large number of enzymes required for the reaction. They also have filamentous metal tolerance, a high binding capacity, and intracellular uptake. Nonetheless, in microorganisms, genetic manipulation to overexpress specific enzymes to increase synthesis is much more difficult. Many studies on the potential use of plant extracts have recently been conducted. Because of their ease of availability, environmental friendliness, and cost-effectiveness, the number of research papers published in this field has increased exponentially over the last decade. Furthermore, plants contain the most effective synthesis compounds, which increase the rate of synthesis. The size and shape distribution of the nanoparticles, as determined by TEM studies, demonstrates that many factors, including the nature of the plant extract, the pH of the solution, and the reaction temperature, influence their morphologies. Obtaining uniform size and shape distribution of Au NCs, however, remains a work in progress.

## Declarations

### Author contribution statement

All authors listed have significantly contributed to the development and the writing of this article.

### Funding statement

This research did not receive any specific grant from funding agencies in the public, commercial, or not-for-profit sectors.

### Data availability statement

No data was used for the research described in the article.

### Declaration of interests statement

The authors declare no conflict of interest.

### Additional information

No additional information is available for this paper.
